# Does disconfirmatory evidence shape safety-and danger-related beliefs of trauma-exposed individuals?

**DOI:** 10.1080/20008066.2024.2335788

**Published:** 2024-04-16

**Authors:** Shilat Haim-Nachum, Tobias Kube, Liron Rozenkrantz, Amit Lazarov, Einat Levy-Gigi, Tanja Michael, Yuval Neria, M. Roxanne Sopp

**Affiliations:** a Department of Psychiatry, Columbia University Irving Medical Center, New York, NY, USA; b New York State Psychiatric Institute, New York, NY, USA; c Department of Clinical Psychology and Psychotherapy, RPTU University of Kaiserslautern-Landau, Landau, Germany; d Azrieli Faculty of Medicine, Bar-Ilan University, Safed, Israel; e School of Psychological Sciences, Tel-Aviv University, Tel Aviv, Israel; f Faculty of Education and the Brain Science Center, Bar-Ilan University, Ramat-Gan, Israel; g Division of Clinical Psychology and Psychotherapy, Saarland University, Saarbrücken, Germany; h Department of Epidemiology, Columbia University Irving Medical Center, New York, NY, USA

**Keywords:** Trauma exposure, belief updating, predictive processing, PTSD symptoms, scenario-based approach, Exposición al trauma, actualización de creencias, procesamiento predictivo, síntomas de TEPT

## Abstract

Recent accounts of predictive processing in posttraumatic stress disorder (PTSD) suggest that trauma-exposed individuals struggle to update trauma-related hypotheses predicting danger, which may be involved in the etiology and maintenance of this disorder. Initial research supports this account, documenting an association between trauma-exposure, impaired expectation updating, and PTSD symptoms. Yet, no study to date has examined biased belief updating in PTSD using a scenario-based approach.

**Objective:** Here, we examined the predictive processing account among trauma-exposed and non-trauma-exposed individuals using a modified Trauma-Related version of the Bias Against Disconfirmatory Evidence task.

**Method:** The task presents both danger-and safety-related scenarios highly relevant for trauma-exposed individuals. For each scenario, participants viewed several explanations and rated their plausibility. Their ability to update their initial interpretation following new-contradictory information was assessed.

**Results:** Preregistered analyses did not reveal any significant findings. Based on indications that our sample may not have been sufficiently powered, we conducted exploratory analyses in an extended sample of participants. These analyses yielded a significant association between reduced belief updating and PTSD symptoms which was evident for disconfirming both safety and danger scenarios. However, the effect sizes we found were in the small-to-medium range.

**Conclusion:** Although preliminary, our current findings support initial evidence that individuals with higher PTSD symptoms show a higher resistance to update their beliefs upon new disconfirmatory evidence. Our results should be interpreted cautiously in light of the extended sample and the limitations of the current study.

## HIGHLIGHTS

- We developed a modified version of the Bias Against Disconfirmatory Evidence task.
- We found significant associations between PTSD symptoms and belief updating.
- The association was evident for disconfirming both safety and danger scenarios.

## Abstract (es)

**Antecedentes:** Relatos recientes sobre el procesamiento predictivo en el Trastorno de Estrés Postraumático (TEPT) sugieren que las personas expuestas a un trauma luchan por actualizar las hipótesis relacionadas con el trauma que predicen el peligro, que puede estar involucradas en la etiología y el mantenimiento de este trastorno. La investigación inicial respalda esta explicación y documenta una asociación entre la exposición al trauma, la alteración de la actualización de las expectativas, y los síntomas de TEPT. Sin embargo, hasta la fecha ningún estudio ha examinado la actualización de creencias sesgadas en el TEPT utilizando un enfoque basado en escenarios.

**Objetivo:** Aquí, examinamos la teoría del procesamiento predictivo entre individuos expuestos y no expuestos al trauma utilizando una versión modificada relacionada con el trauma de la tarea de Sesgo contra la Evidencia No Confirmatoria (BADE, por sus siglas en inglés).

**Método:** La tarea presenta escenarios relacionados tanto con el peligro como con la seguridad, altamente relevantes para las personas expuestas al trauma. Para cada escenario, los participantes vieron varias explicaciones y calificaron su verosimilitud. Se evaluó su capacidad para actualizar su interpretación inicial siguiendo información nueva y contradictoria. Resultados: Los análisis previamente registrados no revelaron ningún hallazgo significativo. Basándonos en indicios de que nuestra muestra podría no haber tenido suficiente potencia, realizamos análisis exploratorios en una muestra ampliada de participantes. Estos análisis arrojaron una asociación significativa entre la reducción de la actualización de creencias y los síntomas de TEPT, que fue evidente al desconfirmar escenarios tanto de seguridad como de peligro. Sin embargo, los tamaños del efecto que encontramos estuvieron en el rango de pequeño a mediano.

**Conclusión:** Aunque preliminares, nuestros hallazgos actuales respaldan la evidencia inicial de que los individuos con mayores síntomas de TEPT muestran una mayor resistencia a actualizar sus creencias ante nueva evidencia no confirmatoria. Nuestros resultados deben interpretarse con cautela a la luz de la muestra ampliada y las limitaciones del estudio actual.

Many individuals worldwide experience at least one traumatic event during their lifetime, with a prevalence of nearly 83% in the USA (e.g. threatened death, severe injury, or sexual violence; Benjet et al., 2016). For some, such trauma exposure results in the development of posttraumatic stress disorder (PTSD; Bryant, 2019; Kessler et al., 2017), which is characterized by intrusive trauma-related memories, hyperarousal, avoidance of trauma reminders, and negative alterations in mood and cognition (American Psychiatric Association, 2013). Yet, for others, PTSD symptoms do not follow. Accordingly, a core question widely addressed in research on PTSD is that of resilience – why do some individuals develop PTSD symptoms following trauma exposure while others do not? (Bryant, 2019; Horn & Feder, 2018; Sayed et al., 2015). One potential answer which seems worth exploring is distortions in *belief updating* – the ways in which individuals adjust their beliefs in light of new information. In the present study, relying on the principles of predictive processing, which were recently applied also to PTSD (Kube et al., 2020; Linson & Friston, 2019; Wilkinson et al., 2017), we aim to examine this possibility.

According to the principles of predictive processing, individuals develop hypotheses about the world based on their prior experiences which are continuously refined as they encounter new sensory input (Wilkinson et al., 2017). Specifically, new information is compared to previously held assumptions, with these assumptions later altered to accommodate this new information when necessary. In the context of traumatic events, this process may alter individuals’ beliefs about the self, others, and the world in a negative way (Bernardi et al., 2019; Brown et al., 2019; Herzog et al., 2021; Kube et al., 2023; Woud et al., 2019). Moreover, given that traumatic events can involve life-threatening situations, it is assumed that these beliefs are assigned a high a-priori likelihood, which results in their persistence, regardless of incoming new disconfirming information (Kube et al., 2020). Thus, the predictive processing framework of PTSD (Kube et al., 2020) proposes that in the aftermath of trauma, individuals struggle to update currently-held trauma-related hypotheses regarding danger. That is, they fail to use new and possibly disconfirmatory evidence to revise existing trauma-related hypotheses. These hypotheses are then consistently activated in everyday life, promoting an excessive sense of continuous threat, which would lead to increased attention allocation to threat-related cues (for a review, see Lazarov et al., 2019). This deficit is hypothesized to facilitate the development of PTSD symptoms following exposure to trauma (Howlett et al., 2021; Kleim et al., 2013; Ter Heide et al., 2017).

Initial research exploring the predictive processing framework of PTSD has shown promising results, revealing an association between exposure to trauma and impaired expectation updating (Haim-Nachum & Levy-Gigi, 2019; 2021), and linking this impairment to PTSD (Sopp et al., 2022). Specifically, in a study with trauma-exposed firefighters, participants observed neutral or trauma-relevant images and completed an updating task associating positive (i.e. gain) or negative (i.e. loss) outcomes with neutral stimuli (i.e. an image of a white door with a symbol on it; Sopp et al., 2022). These associations were then reversed, requiring participants to update the extant stimulus-outcome associations from positive to negative (i.e. a stimulus associated with gain was now associated with loss) or from negative to positive (i.e. a stimulus associated with loss was now associated with gain). That is, when they chose to open the door, they found out if it was associated with a gain or loss. They then had to learn by trial and error to predict the outcome of each door according to its surrounding wall and symbol. Results showed that participants who viewed traumatic – as compared to neutral – images prior to the updating task showed reduced updating, which specifically manifested in difficulties to update the negative stimulus-outcome associations, with this deficit positively associated with PTSD symptoms. While these findings support the assumption that traumatic content is more resistant to updating, their generalizability to real-life trauma is limited. Previous research focused on the field of dysfunctional beliefs in PTSD but no studies investigated how these beliefs are updated (Elwood et al., 2007; White et al., 2008). Belief updating has only been investigated in partial reversal paradigms which assess beliefs regarding experimental stimuli, whereas beliefs in the real world are much more complex. Hence, effectively testing the relationship between belief updating and PTSD requires a task that more closely resembles real-world experiences.

A more ecological-valid task to assess belief updating processes is the Bias Against Disconfirmatory Evidence (BADE; Woodward et al., 2006). In the task, participants are shown a series of scenarios. For each scenario, they read three statements and view four explanations that might account for the known facts in this particular situation. Participants are asked to rate these interpretations’ plausibility as the scenarios unfolded. Their ability to change their confidence of these interpretations in line with new contradictory information reflects flexible belief-updating. The ecological validity of this task is reflected in the inclusion of common real-life situations and the assessment of belief updating processes that mirror those in everyday life. The task tracks the dynamic, step-by-step revision of new information that is depicted in a new light, allowing a more nuanced exploration of the processes that guide interpretation of novel evidence that matches or does not match one’s initial beliefs. In our modified Trauma-Related version of the BADE task (TR-BADE), we included two scenario types: 1) disconfirming-danger scenarios, which initially implied danger but eventually had a neutral ending (e.g. a stranger increasing his pace behind you before he ultimately reveals himself to be running towards the bus stop up ahead); and 2) disconfirming-safety scenarios, which initially implied safety but gradually unfolded in a potentially dangerous way (e.g. an approaching dog seems friendly at a distance but appears more aggressive as it draws closer). Prior studies have used an emotional version of the BADE task to test bias and inflexibility in the interpretation of unfolding ambiguous situations, in depression and social anxiety (Everaert et al., 2018). It was found that if the scenario initially suggested a negative interpretation, individuals with either symptoms of depression or anxiety struggled to update their beliefs and abandon that interpretation, even when new positive information was introduced. Further, the severity of these symptoms was associated with difficulties to update these beliefs (Everaert et al., 2018). Yet, to the best of our knowledge, no study to date has examined belief updating in PTSD using a scenario-based approach such as the BADE task.

Using the TR-BADE task, we compared belief updating in trauma-exposed (TE) vs non-trauma-exposed control (NT) individuals. We hypothesized that TE, as opposed to NT individuals, would show reduced belief updating for scenarios that disconfirmed an initial sense of danger (Hypothesis #1a) and enhanced belief updating for scenarios that disconfirm an initial sense of safety (Hypothesis #1b). Moreover, we predicted that the number of trauma types experienced would be negatively associated with belief updating for scenarios that disconfirmed an initial sense of danger in TE individuals (Hypothesis #2a) and positively correlated with belief updating for scenarios that disconfirmed an initial sense of safety in these individuals (Hypothesis #2b). Additionally, we predicted that PTSD symptom severity would be negatively associated with belief updating for scenarios that disconfirmed an initial sense of danger in TE individuals (Hypothesis #3a) and positively correlated with belief updating for scenarios that disconfirmed an initial sense of safety in these individuals (Hypothesis #3b). The decision to analyze the number of trauma types experienced and PTSD symptom severity through separate hypotheses is based on the recognition that these two variables may not necessarily align in a straightforward manner. It is possible for individuals to have experienced numerous traumatic events without manifesting (severe) trauma symptoms. By formulating distinct hypotheses for each variable, we aimed to explore the potential independent contributions of both variables to belief updating processes in trauma-exposed individuals. In addition, we sought to explore individuals’ performance of belief updating for scenarios that disconfirmed an initial sense of safety, to assert whether this phenomenon reflects a more general cognitive tendency or is distinctly associated with danger-related stimuli.

## 1. Methods

### 1.1. Participants

We recruited 232 Israeli participants via the online platform *Ipanel*. We initially sought to recruit 154 participants (77 female) with TE (Life Events Checklist for DSM-5; LEC-5; Weathers, Blake, et al., 2013, score ≥ 1, excluding Item 17) and 78 participants (39 female) without TE (LEC-5 = 0, excluding Item 17). A dichotomous question was used to determine group assignment (‘Have you ever been exposed to one or more of the following events?’), followed by 16 traumatic events from the LEC-5 as examples. Subsequently, during the data analysis phase, we discovered that some of the participants who were originally labelled as non-trauma-exposed control (NT) individuals nevertheless reported experiencing indirect forms of trauma exposure (i.e. witnessing a traumatic event) adhering to DSM-5 Criterion A of trauma exposure. Hence, we had to adjust the group quotas, which resulted in 189 TE participants (93 female) and 43 NT participants (23 female). Using G*Power software (Faul et al., 2007), the sample size to test Hypothesis #1 was based on the detection of a small-to-medium-sized difference (*d* = .35; one-sided) between the TE and NT group, with a power of 0.80 and an allocation ratio of 2:1. We chose a 2:1 ration because we were interested in analyzing regression analyses only in the TE group. The sample size to test Hypotheses #2 and 3 was based on the detection of a small-to-medium-sized association (*r* = .20; one-sided) between trauma exposure and updating, with a power of 0.80. To assess whether effects were driven by individuals with extreme values, we conducted sensitivity analyses, excluding participants with BADE values that exceeded 3.0 interquartile ranges above the upper or below the lower quartile.

Eligible participants were at least 18 years old with sufficient reading skills and understanding of the local language. Participants were excluded if they: 1) missed out on answering more than 20% of each experimental condition; 2) constantly rated the absurd interpretations most plausible; 3) failed to notice attention checks implemented throughout the experiment (see below). A total of 11 participants were excluded based on these criteria. The final analysis sample comprised 221 participants (52% female, *M*age = 46.06, *SD*age = 14.74; see Table 1 for detailed sample characteristics).

**Table 1.** Demographic and psychometric characteristics for TE and NT individuals (*N* = 221) in Study 1 and TE individuals (*N* = 163) in Study 2.

	Study 1			Study 2
Variables	Trauma-exposed (*n* = 180) *M* (*SD*)	Non-trauma exposed (*n* = 41) *M* (*SD*)	*Significance*	Trama-exposed (*n* = 163) *M* (*SD*)
Age (years)	47.11 (14.79)	44.12 (15.32)	*p* = .25	48.33 (14.33)
Female/Male	92/88	23/18	*p* = .57	82/81
Education (years)	15.04 (2.71)	14.46 (2.55)	*p* = .21	15.68 (2.81)
PCL-5	11.39 (13.24)	—	***p* = .00**	12.34 (13.61)
PHQ-9	4.55 (4.5)	2.71 (3.59)	***p* = .01**	5.47 (4.51)
LEC-5	4.14 (2.58)	0	***p* = .00**	*** ***
STAI State	37.33 (12.4)	33.83 (9.12)	***p* = .04**	*** ***
STAI Trait	36.63 (12.15)	33.24 (10.98)	*p* = .10	* *
TR-BADE_DD	6.94 (3.67)	6.21 (3.33)	*p* = .24	4.42 (2.7)
TR-BADE_DS	8.86 (4.06)	8.1 (4.58)	*p* = .29	6.83 (3.62)
CRT	0.67 (0.92)	0.46 (0.92)	*p* = .19	* *

Note. The values for Female/Male and households represent frequencies. PCL-5 = PTSD checklist for DSM-5; PHQ-9 = depressive symptoms; LEC-5 = life events checklist; STAI – anxiety scores; TR-BADE_DD = disconfirming danger scenarios; TR-BADE_DS = disconfirming safety scenarios; CRT = Cognitive Reflection Test.

### 1.2. Measures

*Trauma exposure.* Trauma exposure was assessed using the Life Events Checklist (LEC; Weathers, Blake, et al., 2013), which screens for 16 traumatic events. For each event, responders indicated whether the event had directly happened to them; whether they had witnessed it happen to someone else; whether they had learned about it happening to a person close to them; whether they were exposed to it as part of their job; or whether none of these applied to them. In addition, participants were asked how strongly they feel that any traumatic event(s) that they have experienced impacted their current life.

*PTSD Symptoms.* PTSD symptoms were assessed with the Posttraumatic Stress Disorder Checklist (PCL-5; Weathers, Litz, et al., 2013) – a 20-item questionnaire that assesses the DSM-5 symptoms of PTSD over the past month. Participants completed the PCL-5 in relation to the worst traumatic event from the LEC. Responses are scored on a five-point scale ranging from 0 = ‘not at all’ to 4 = ‘extremely’. Forty-one (12.2%) participants met the clinical threshold (PCL Sum scores ≥31; based on Bovin et al., 2016). This is a sound measure that has strong psychometric properties, including convergent and discriminant validity and test-retest reliability (Blevins et al., 2015). Internal consistency in the current study was .91.

*Depression.* Depression was assessed using the *Patient Health Questionnaire-9* (PHQ-9; Kroenke et al., 2001), a brief instrument assessing nine depressive symptoms experienced over the past two weeks (e.g. anhedonia, sleep disturbances, low self-esteem, concentration difficulties). Responses are scored from 0 =  ‘not at all’ to 3 = ‘nearly every day’. Symptom severity is indicated by the sum of all item scores. The psychometric properties of this measure proved to be adequate with a robust factor structure and good consistency (e.g. Krause et al., 2010; Richardson & Richards, 2008). Internal consistency in the current study was .86. Due to the frequent comorbidity of PTSD and depression in trauma-exposed individuals (Flory & Yehuda, 2015), and updating biases in depression (for a review, see Kube, 2023) we sought to control this variable in our analyses.

*Anxiety.* The State-Trait Anxiety Inventory (STAI; Spielberger et al., 1983) is a 40-item questionnaire designed to measure two dimensions of anxiety, namely, trait (STAI-T) and state anxiety (e.g. a transient emotional state; STAI-S). Items are rated on a 4-point scale ranging from 1 =  ‘not at all’ to 4 =  ‘totally,’ for total scores of 20–80, with higher scores indicating higher trait and state anxiety levels. The cutoff for a clinically-significant anxiety state and trait is between 39 and 40. This measure has shown good test-retest reliability coefficients and good concurrent validity (Spielberger, 1989; Spielberger et al., 1983). Internal consistency in the current study was .94 for each state and trait anxiety.

*Cognitive Reflection.* The Cognitive Reflection Test (CRT; Frederick, 2005) is a 3-item questionnaire that measures cognitive processing, specifically the tendency to suppress an incorrect intuitive answer for a more deliberate, correct one. An example item is ‘If it takes 5 machines 5 min to make 5 widgets, how long would it take 100 machines to make 100 widgets?’. While the intuitive answer is 100 min, the correct answer – which requires more reflection and conscious thought – is five minutes. The measure is scored as the total number of correct answers (range: 0–3). Despite its brevity, the CRT is widely employed in the literature as a reliable tool for assessing cognitive reflection abilities (Barr et al., 2015; Campitelli & Gerrans, 2014; Primi et al., 2014). By including this test as a covariate in our analyses, we aimed to isolate the unique association between belief updating and PTSD symptoms, independent of general cognitive processing differences. This measure was recently found to predict performance on biases tasks (Toplak et al., 2011).

Sociodemographic variables, including age, gender, education, country of birth, language proficiency, family status, employment status, and religious affiliation were assessed using a brief self-report questionnaire.

### 1.3. Trauma-Related version of the BADE Task (TR-BADE)

The main dependent variable was belief updating on the TR-BADE task. Similar to the original task version (Woodward et al., 2006), participants were shown a series of scenarios that involved situations they could encounter in their daily lives, and were asked to imagine the events as if they were experiencing them firsthand or seeing them through their own eyes. They then read three statements containing further information about each scenario and viewed several explanations that may account for these additional details.

The TR-BADE task included 24 novel scenarios of common danger/safety-related situations that participants could encounter in their lives. Two scenario types were developed, each comprised of eight scenes, in addition to eight control scenarios. The disconfirming danger scenario type initially implied danger (Statements 1 and 2) but eventually had a neutral ending (Statement 3). For example, Statement 1 might read: ‘It is late at night, and you are walking through a park. You can hear a stranger walking behind you. You suddenly get the feeling that he is following you’. The sense of danger was then strengthened by Statement 2: ‘As you start to walk faster, the stranger’s steps start to become faster as well’. The scenario was then resolved with a neutral ending, when participants learned that ‘a bus is approaching, and the stranger started running towards the station ahead’ (Statement 3). This type of scenario was inspired by potentially traumatic events that are especially common in the Israeli routine (e.g. living in conflict zones, experiencing frequent terror attacks). The second scenario type, Disconfirming safety scenarios, included scenarios that initially implied safety/neutral situations that ended more negatively in a way that implies on a trauma-related ending. For example, here, Statements 1 and 2 may read: ‘It is a warm summer day, and you are taking your dog for a walk. You can see another dog at the end of the road approaching you’ and ‘The approaching dog is a friendly dog from the neighborhood,’ respectively, with Statement 3 stating that ‘The approaching dog is flashing its teeth and darts towards you.’ These two scenario types are inherently trauma-related; the disconfirming danger scenarios initiate with a dangerous/trauma-related setting and are subsequently disconfirmed by more neutral/safe information, while the disconfirming safety scenarios initiate with safe/neutral settings and are disconfirmed by more dangerous, trauma-related information. The distinction between them lies in the direction of the outcomes. Both scenario types were randomized and counterbalanced across participants. The eight control scenarios included four control scenarios for each valence (i.e. confirming danger and confirming safety), and did not require any disconfirmation (i.e. scenarios that initially implied safety had a neutral ending, and vice versa). We included control scenarios to make the contradicting statement (i.e. Statement 3) in the disconfirmig danger and disconfirming safety scenario types less predictive. Hence, these trials were not included in data analyses. Four researchers at a post-doctoral level carefully designed and reviewed the scenarios. The researchers are experienced in the field of updating and some of them – born and raised in Israel – are well-familiar with stressful events that often occur in Israel.

As in the original BADE task, following each statement, participants were required to rate the plausibility of four interpretations of the presented scenario on a 21 point-scale ranging from ‘poor’ ( = 1) to ‘excellent’ ( = 21). These interpretations were classified as either *absurd* – they described interpretations that remained impossible from the first statement through the resolution of the scenario in Statement 3 (e.g. in the example of a stranger increasing his pace behind you, an absurd item would be: ‘the stranger is planning to hand you an ice cream cone’); *lure* – they were initially the most plausible option but became less plausible after the third statement (two different lures were presented alongside each scenario, e.g. ‘The stranger has a gun and plans to mug you’; or ‘The stranger is planning to attack and batter you’); and *true* – they were initially less plausible than the lures but became the most plausible option after Statement 3 (e.g. ‘The stranger is going on a bus’). The order of the appearance of these interpretations was randomized across statements and participants. The main outcome (BADE score) reflects the mean change in confidence (i.e. plausibility rating) from sentence two to sentence three for the lure interpretations, reflecting flexible updating. For example, a score of eight in disconfirming danger scenarios indicates a decrease in plausibility rating from 18 to 10 from statement 2 to statement 3.

### 1.4. Procedure

All study procedures were approved by the Saarland University’s Institutional Review Board (#22–01) in accordance with the Declaration of Helsinki. The study was pre-registered in January 2022 (available at https://aspredicted.org/D2C_DST). The data were collected online using the software Qualtrics (Provo, USA) over two weeks in February 2022. The study was described as a research project on ‘the relationship between trauma exposure and interpretation of events’. Following informed consent participants first completed a demographic questionnaire, followed by the TR-BADE task, and measures assessing trauma exposure, psychopathology, and cognitive reflection. No identifying information was collected. To ensure data quality, we programmed attention checks throughout the experiment requiring participants to select a specific answer. For example, in a multiple-choice question ‘We want to test your attention, please click on the answer *Agree*’, those who selected other options (e.g. *Disagree, Strongly Agree*) were considered inattentive. The study lasted for about 40 min, and participants were given approximately 40 credit points that were translated to money/gift cards using Ipanel service.

### 1.5. Data Analysis

Data analysis was performed using IBM SPSS Statistics 25, R, and JASP. The Type-I error level was set to .05 for all analyses. Degrees of freedom can vary due to missing data. Prior to conducting analyses, we examined the distribution of all study variables for potential outliers (data points that exceeded 3.0 interquartile ranges above the upper or below the lower quartile of the distribution). No outliers were detected. Note that analyses were conducted as one-sided as we had clear hypotheses about the direction of the effects on the findings (See Böschen, 2023; Lakens et al., 2018).

To test Hypothesis #1, we conducted a mixed analysis of variance (ANOVA) including Trauma Exposure (TE vs. NT) as a between-subjects factor, and Disconfirmation Type (for Disconfirming danger vs. Disconfirming safety scenarios) as the within-subjects factor. The BADE score served as the outcome measure. Significant interaction effects were followed up by conducting *t*-tests. Partial η^2^ was calculated to illustrate effect sizes.

To test Hypothesis #2 and #3, we conducted hierarchical linear regression analyses with the BADE score (for Disconfirming danger and Disconfirming safety scenarios, respectively) as the dependent variable. For both analyses, in the first step, all control variables (gender, depression symptoms, and cognitive reflection scores) were simultaneously entered into the model to account for any variance explained by these variables. For Hypothesis #2 the number of trauma types (measured using the LEC-5) was entered as a predictor in the second step, while for Hypothesis #3 severity of PTSD symptoms (PCL-5 scores) was entered as a predictor in Step 2. Standardized regression coefficients (*β*) with *t*-values as well as overall model tests are reported. Effect sizes are illustrated in terms of the amount of variance accounted for by each model (adjusted *R*²).

## 2. Results

### 2.1. Change of plausibility ratings across scenarios

**Figure 1.**
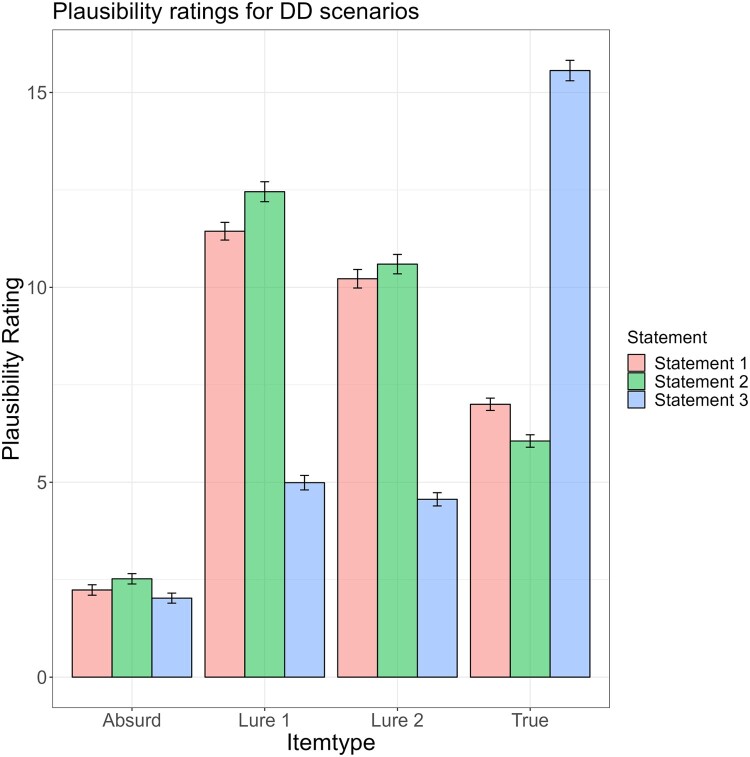
Means and standard errors of plausibility ratings during disconfirming danger scenarios for true interpretations, absurd interpretations and lures.

**Figure 2.**
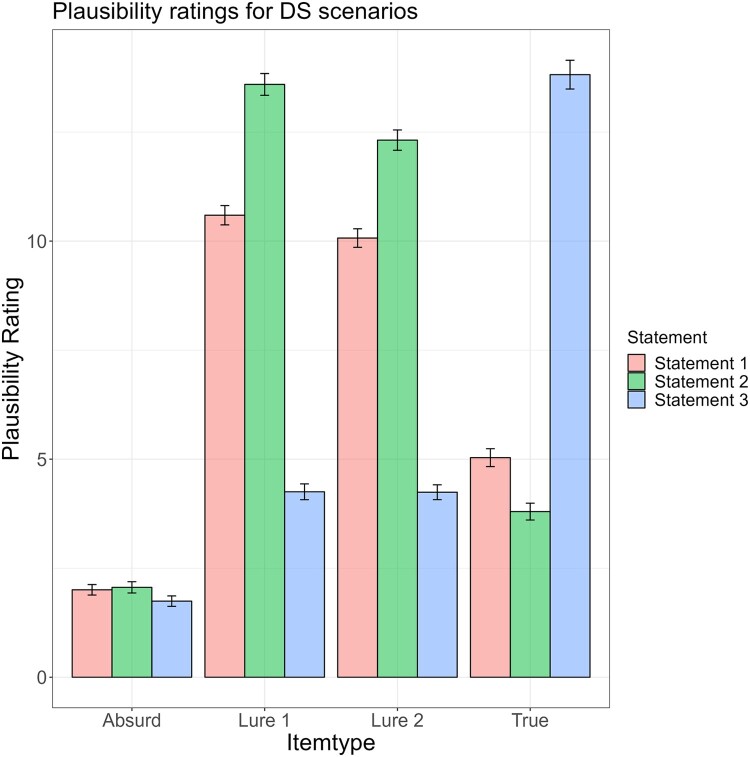
Means and standard errors of plausibility ratings during disconfirming safety scenarios for true interpretations, absurd interpretations and lures.

The change of mean plausibility ratings across disconfirming danger and disconfirming safety scenarios is illustrated in Figures 1 and 2 and Table 2. As expected, plausibility ratings for Lure 1 and 2 were found to increase from Statement 1 to Statement 2 and decrease from Statement 2 to Statement 3. By contrast, plausibility ratings for the true interpretations were found to decrease from Statement 1 to Statement 2 and increase from Statement 2 to Statement 3. Plausibility ratings for absurd interpretations remained at a constantly low level.

**Table 2.** Mean plausibility ratings in the BADE task.

		Disconfirming-danger scenarios					Disconfirming-safety scenarios				
Item type	Statement	Mean	Std. Error	95% Confidence Interval		Significance	Mean	Std. Error	95% Confidence Interval		Significance
				Lower Bound	Upper Bound				Lower Bound	Upper Bound	
Lure 1	1	11.440	.227	10.993	11.887		10.594	.221	10.159	11.030	
	2	12.454	.256	11.949	12.960	1 < 2: *p* = .001	13.593	.250	13.100	14.086	1 < 2: *p* < .001
	3	4.989	.186	4.622	5.356	2 > 3: *p* = .001	4.252	.181	3.895	4.608	2 > 3: *p* < .001
Lure 2	1	10.221	.238	9.751	10.690		10.070	.213	9.651	10.490	
	2	10.596	.249	10.105	11.086	1 < 2: *p* = .001	12.316	.233	11.857	12.776	1 < 2: *p* < .001
	3	4.562	.170	4.227	4.896	2 > 3: *p* = .001	4.242	.170	3.906	4.578	2 > 3: *p* < .001
Absurd	1	2.236	.134	1.973	2.499		2.005	.120	1.770	2.241	
	2	2.523	.133	2.260	2.785	1 < 2: *p* = .001	2.061	.129	1.806	2.316	1 < 2: *p* < .309
	3	2.027	.129	1.772	2.282	2 > 3: *p* = .001	1.745	.120	1.508	1.982	2 > 3: *p* < .001
True	1	7.000	.158	6.689	7.312		5.035	.205	4.631	5.438	
	2	6.058	.160	5.743	6.374	1 > 2: *p* = .001	3.798	.193	3.417	4.179	1 > 2: *p* < .001
	3	15.562	.262	15.046	16.078	2 < 3: *p* = .001	13.817	.330	13.166	14.469	2 < 3: *p* < .001

Note. Std. Error = Standard Error.

### 2.2. Associations between trauma exposure and belief updating

A significant main effect of Scenario Type emerged, *F*(1,219) = 60.23, *p* < .001, *η_p_² *= .216, reflecting higher BADE scores – that is, stronger updating – for Disconfirming safety scenarios as opposed to Disconfirming danger scenarios, *t*(220) = 10.06, *p* < .001. None of the other main or interaction effects reached significance (all *p-*values > .230). Secondary analyses conducted to explore the existence of group differences for the control scenarios similarly did not reveal a significant group effect or group-related interactions. In line with the main analyses, participants showed a stronger tendency to confirm safety-related than danger-related scenarios.

### 2.3. Regression analyses

#### 2.3.1. Associations between trauma exposure and belief updating in TE individuals

Including LEC scores as an independent variable in Step 2 did not add to the explanation of variance in belief updating, neither for the Disconfirming danger scenario type, *ΔR²* = .001, *F*(1,172) = 0.22, *p* = .643, nor for the Disconfirming safety scenario type, *ΔR²* = .001, *F*(1,172) = 5.56, *p* = .628 (see Tables 3 and 4).

**Table 3.** Hierarchical regression analyses for disconfirming **danger scenarios** in traumatized individuals, *n* = 177 (extended sample; *n* = 343).

	Baseline model			+ LEC-5			+ PCL-5		
Predictors	Estimates	CI	*p*	Estimates	CI	*p*	Estimates	CI	*p*
(Intercept)	8.55 (8.75)	6.73–10.36 (7.44 –10.06)	**<.001** **(<.001)**	8.40 (8.77)	6.48–10.32 (7.40 –10.14)	**<.001** **(<.001)**	8.72 (8.76)	6.89–10.54 (7.46 –10.06)	**<.001** **(<.001)**
PHQ-9	−0.04 (−0.01)	−0.17–0.08 (−0.09–0.08)	.482 (.868)	−0.05 (−0.01)	−0.18–0.08 (−0.09–0.08)	.433 (.889)	0.04 (0.09)	−0.14–0.21 (−0.03–0.21)	.677 (.152)
Gender	−0.81 (−1.22)	−1.91–0.29 (−1.99 – −0.46)	.148 (**.002**)	−0.83 −1.22	−1.94–0.28 (−1.99 – −0.44)	.140 **(.002**)	−0.84 (−1.19)	−1.94–0.26 (−1.95 – −0.42)	.132 (**.002**)
CRT	−0.35	−0.96–0.25	.250	−0.36	−0.97–0.24	.239	−0.41	−1.02–0.20	.183
LEC-5				0.05 (−0.01)	−0.17–0.27 (−0.16–0.14)	.643 (.917)			
PCL-5							−0.04 (−0.04)	−0.10–0.02 (−0.08–0.00)	.188 (**.028**)
R^2^ / R^2^ adjusted	0.024 / 0.007 (0.028 / 0.022)			0.026 / 0.003 (0.028 / 0.020)			0.034 / 0.012 (0.042 / 0.034)		

Note. Excluding CRT scores from the main analyses (*n *= 180) did not change the overall pattern of the results; PHQ-9 = depressive symptoms; CRT = Cognitive Reflection Test; LEC-5 = life events checklist; PCL-5 = PTSD checklist for DSM-5.

**Table 4.** Hierarchical regression analyses for disconfirming **safety scenarios** in traumatized individuals, *n* = 177 (extended sample *n* = 343).

	Baseline model			+ LEC-5			+ PCL-5		
Predictors	Estimates	CI	*p*	Estimates	CI	*p*	Estimates	CI	*p*
(Intercept)	10.53 (11.51)	8.52–12.54 (9.99–13.02)	**<.001** **(<.001)**	10.70 (11.66)	8.57–12.83 (10.08–13.25)	**<.001** **(<.001)**	10.72 (11.52)	8.69–12.75 (10.01–13.02)	**<.001** **(<.001)**
PHQ-9	−0.12 (−0.10)	−0.26–0.01 (−0.20 – −0.00)	.079 **(.043)**	−0.12 (−0.09)	−0.26–0.03 (−0.19–0.01)	.107 (.068)	−0.03 (0.01)	−0.22–0.16 (−0.12–0.15)	.747 (.832)
Gender	−0.73 (−1.33)	−1.95–0.50 (−2.21 – −0.44)	.243 **(.003)**	−0.70 (−1.29)	−1.93–0.53 (−2.18 – −0.39)	.260 **(.005)**	−0.76 (−1.28)	−1.98–0.46 (−2.16 – −0.40)	.220 **(.005)**
CRT	−0.05	−0.72–0.62	.882	−0.04	−0.71–0.64	.910	−0.12	−0.80–0.56	.731
LEC-5				−0.06 (−0.06)	−0.30–0.18 (−0.23–0.11)	.628 (.503)			
PCL-5							−0.05 (−0.06)	−0.11–0.02 (−0.10 – −0.01)	.177 **(.018)**
*R*^2^ / *R*^2^ adjusted	0.026 / 0.009 (0.034 / 0.028)			0.027 / 0.005 (0.035 / 0.027)			0.036 / 0.014 (0.050 / 0.041)		

Note. Excluding CRT scores from the main analyses (*n* = 180) did not change the overall pattern of the results; PHQ-9 = depressive symptoms; CRT = Cognitive Reflection Test; LEC-5 = life events checklist; PCL-5 = PTSD checklist for DSM-5.

#### 2.3.2. Associations between PTSD symptoms and belief updating in TE individuals

Including PCL scores as an independent variable in Step 2 did not incrementally explain variance in belief updating, neither for the Disconfirming danger scenario, *ΔR²* = .010, *F*(1,172) = 1.75, *p* = .188, nor for the Disconfirming safety scenario, *ΔR²* = .010, *F*(1,172) = 1.84, *p* = .177 (see Tables 3 and 4).

#### 2.3.3. Follow-up analyses: Extended participant sample

While our analyses did not reveal any significant association between PCL scores and belief updating, regression weights indicated a trend towards a negative, yet small effect size (Disconfirming danger: *β* = −.15; Disconfirming safety: *β* = −.14). Given that our study was powered to find a larger effect size (*β *= .20), its sample size may have been underpowered to detect any associations between PCL scores and belief updating. In order to test this possibility, we used data from a separate Ipanel sample of TE individuals (*N* = 163; for sample characteristics see Table 1), who underwent the same study procedure and completed the same TR-BADE task as well as the LEC-5, PCL-5 and PHQ-9. The data were collected as part of a different registered study (for more details, see https://osf.io/sabxz) on the role of belief updating in the relationship between reward processes and psychopathology in TE individuals and included the same type of sample (Israeli participants). We combined both samples (total *N* = 343) and repeated the analyses to test Hypothesis #3 (see Tables 3 and 4).

**Figure 3.**
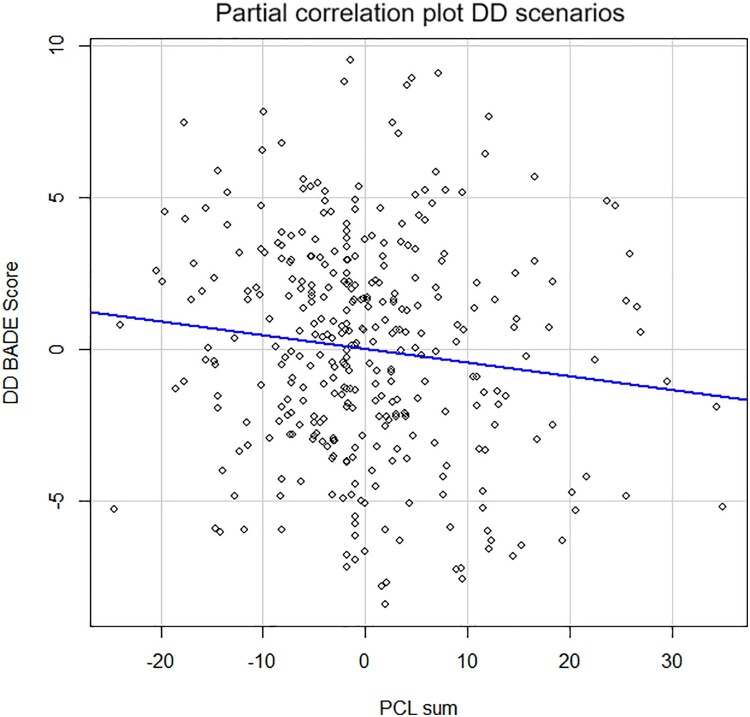
Partial regression plot for the association between PCL-5 scores (range  = 0–64, *M *= 11.84, *SD *= 13.41) and the BADE score for disconfirming danger (DD) scenarios.

In this extended sample, PCL scores were found to significantly add to the explained variance in belief updating for Disconfirming danger scenarios beyond the baseline model, *ΔR²* = .014, *F*(1,339) = 4.88, *p* = .028. As hypothesized, higher levels of PTSD symptoms predicted reduced updating, *β* = −.17, *t*(339) = 2.21, *p* = .028 (see Figure 3). That is, more symptomatic individuals were less able to update their initial beliefs regarding danger even when provided with disconfirmatory neutral/safe information.

**Figure 4.**
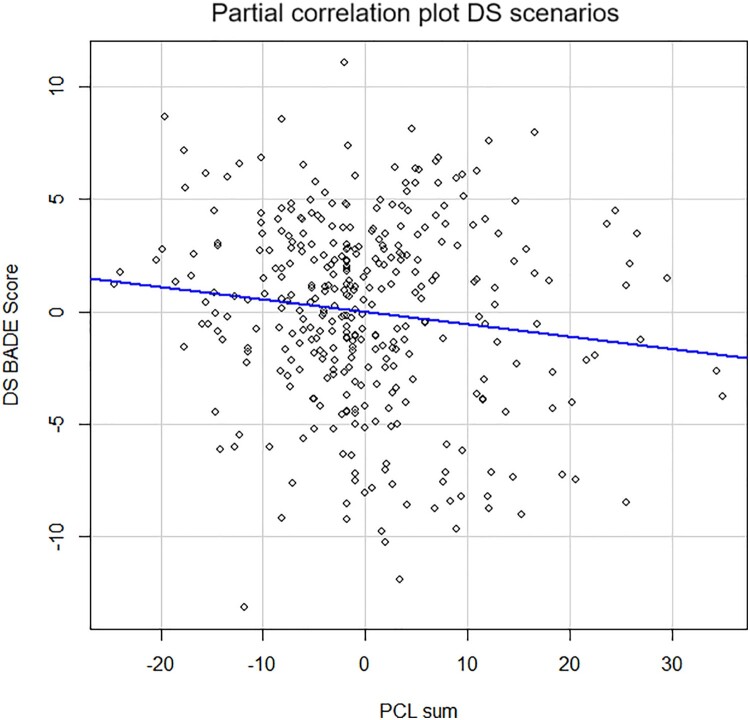
Partial regression plot for the association between PCL-5 scores (range  = 0–64, *M *= 11.84, *SD *= 13.41) and the BADE score for disconfirming safety (DS) scenarios.

PCL-5 scores were further found to improve the prediction of belief updating for Disconfirming safety scenarios beyond the baseline model, *ΔR²* = .016, *F*(1,339) = 5.61, *p* = .018. Contrary to our prediction, here, higher levels of PTSD symptoms were associated with reduced updating also for Disconfirming safety scenarios, *β* = −.18, *t*(339) = 2.37, *p* = .018. That is, more symptomatic individuals were less able to update their initial beliefs regarding safe situation even when provided with disconfirmatory dangerous/potentially traumatic information (see Figure 4).

No significant results emerged for associations between LEC-5 scores and belief updating (see Tables 3 and 4).

## 3. Discussion

The current study examined the core assumptions of the predictive processing framework of PTSD using a scenario-based approach. Contrary to our hypotheses, TE individuals were not found to show any differences in belief updating for Disconfirming danger and Disconfirming safety scenarios, compared to no trauma exposed controls. In addition, our regression analysis showed that the number of trauma types was not predictive of belief updating in either scenario. While PTSD severity initially showed similar null results, increasing the sample size based on the second power analysis based on the observed effect size of the initial analysis revealed a number of significant findings. Specifically, PTSD severity was found to be predictive of belief updating for the Disconfirming danger scenario type, echoing our predictions, but also for the Disconfirming safety scenario type, contrary to our hypothesis. That is, PTSD severity was associated with reduced belief updating also for scenarios that start as safe and become dangerous, indicating a more general belief updating deficit.

Our finding that trauma exposure was not associated with belief updating contrasts with our hypothesis as well as with previous findings suggesting that exposure to traumatic – as opposed to neutral – material alters updating of negative outcome expectations (Sopp et al., 2022). This could be attributed to differences between study designs since we contrasted updating performance between TE and NT individuals whereas our previous study contrasted updating performance for traumatic as compared to neutral material in TE individuals. However, several studies have found a similar updating deficit (Sopp et al., 2022) in TE as opposed to NT individuals (Croft et al., 2022; Levy-Gigi & Richter-Levin, 2014; Levy-Gigi et al., 2014). One important factor that may account for this result discrepancy is the ecological validity of the task used to assess belief updating. While the present study used a scenario-based approach to approximate updating in real life, previous studies examined belief updating through experimental learning tasks using highly standardized stimuli (e.g. symbols or beads), which enables tapping into the process of information processing rather than focusing only on the output of that process. Hence, while the present approach does yield greater ecological validity, it might lack the ability to detect the effects of trauma exposure on belief updating. Another important consideration is the homogeneity of the study sample. While the current study used a rather heterogenous sample of individuals exposed to various types of traumatic events in different intensities, past research used more homogeneous samples of TE individuals (e.g. first-responders), with some studies further finding that updating deficits differ even between different subpopulations (e.g. firefighters vs. police officers) (Levy-Gigi & Richter-Levin, 2014).

The second aim was to explore links between PTSD symptom severity and belief updating in TE individuals. Overall, our findings indicate that an association is present, yet smaller than anticipated. For disconfirming danger scenarios, we were able to demonstrate a negative link between PTSD symptom severity and belief updating, aligning with previous cognitive models, suggesting that interpretation biases are a maintaining factor of PTSD symptoms (Amir et al., 2002; Ehlers & Clark, 2000). Importantly, this finding supports the predictive processing framework, which assumes that TE individuals have strong trauma-related perceptual hypotheses that are highly resistant to updating by disconfirmatory evidence, offering nuanced insights into the nature of belief-updating processes and their role in the etiology of PTSD. This notion also converges with Foa and colleagues’ conceptualization of PTSD as a failure of natural recovery (Foa & McLean, 2016). Natural recovery is assumed to occur when trauma survivors re-engage with traumatic stimuli, while experiencing the absence of feared consequences. The predictive processing framework makes an important addition by highlighting that these experiences will only lead to natural recovery if they result in updating of trauma-associated hypotheses (or fear structures in the model of Foa & Kozak, 1986). This may be reflected in our finding that participants, who struggled to use disconfirmatory evidence to update their danger-related hypotheses also reported greater PTSD symptom severity.

For the Disconfirming safety scenario type, we initially hypothesized that those with higher PTSD symptom severity would show stronger updating of positive expectations in the light of disconfirming, dangerous information. However, we found a negative association between PTSD symptom severity and belief updating for Disconfirming safety scenarios, reflecting less revising of initial positive expectations. This suggests a tendency for individuals with higher PTSD symptoms levels to maintain positive beliefs even in the presence of disconfirmatory evidence, potentially leading to the possibility of re-entering dangerous situations and increasing the risk for further traumatization. Similar mechanisms have been assumed to account for revictimization in survivors of childhood trauma (DePrince, 2005; Gobin & Freyd, 2009). Since our measure of trauma exposure bears several limitations, though, caution is warranted in drawing strong conclusions in this regard. However, future studies may aim to dissect the extent to which a reduced tendency to update Disconfirming safety scenarios in those with high PTSD severity could be linked to repeated trauma exposure, particularly in the interpersonal domain. Overall, the effect sizes we found were in the small-to-medium range and significant results only emerged after extending the originally planned and preregistered sample with an additional sample. Though this seems to indicate limited clinical significance (Kraemer et al., 2003), it is important to note that we used a scenario-based approach to assess belief updating. We believe that effects may be considerably larger if assessed in real-world settings, which should be tested in future studies (Feldmann et al., 2023; Kube et al., 2022; Ossola et al., 2020). Relatedly, clinical significance is challenged by the fact that the majority of our sample did not meet the PTSD threshold. Future studies involving clinical populations are thus essential to validate our findings. If clinical significance is confirmed, interventional research should focus on investigating strategies explicitly aimed at enhancing belief updating in individuals struggling with the persistent impact of trauma.

Finally, our regression analyses revealed a substantial gender effect that warrants attention. Specifically, females reported higher levels of exposure to types of traumatic events and demonstrated higher levels of PTSD symptoms compared to males. This observed gender disparity aligns with previous research highlighting sex differences in the prevalence of trauma and vulnerability to PTSD (Blanco et al., 2018; Olff, 2017).

The cross-sectional design of our study constitutes one of the study’s limitations. Since belief updating and symptoms were assessed post-trauma and simultaneously, we cannot make causal inferences on the relationship between belief updating and PTSD symptom severity. Given that prospective study designs require extensive resources, the current study aimed to provide a preliminary assessment of correlational associations to pave the way for further, more comprehensive research. Another limitation is our operationalization of belief updating. That is, we chose to use a scenario-based approach rather than assessing updating real-life beliefs (Feldmann et al., 2023; Kube et al., 2022; Ossola et al., 2020). Moreover, we inadvertently under sampled non-exposed participants and may have been underpowered to detect the effects of trauma exposure. While we were able to test the correlational association between trauma exposure and updating performance in our extended sample, the LEC-5 does not assess the number of times a specific event has happened. Hence, our measure of trauma exposure may not have been sensitive enough to detect correlational associations. Finally, several limitations arise from our study sample and sampling strategy. First, the data quality of online panels has been criticized recently (Kees et al., 2017). However, Chmielewski and Kucker (2020) suggest that detrimental effects can be mitigated by using response validity indicators and screening the data, which we implemented by using attention checks and excluding participants who consistently rated absurd explanations as most plausible. Nevertheless, data quality may be reduced in our sample, potentially reflected in the fact that our prescreening strategy did not result in the desired ratio of TE vs. NT participants. However, we believe that these constraints would likely mitigate finding existing effects (due to noisy data) rather than resulting in false-positive findings. Moreover, we conducted our study in an Israeli sample of participants, who experience a high rate of trauma exposure due to the continuous occurrence of terror and missile attacks on the general population (Diamond et al., 2010; Lahad & Leykin, 2010). Hence, generalizability to other populations may be limited. Moreover, the current sample did not have high PTSD symptom levels with most of the participants reporting sub-clinical symptoms, which might explain the limited findings. Future studies may benefit from recruiting individuals in the clinical range and applying the TR-BADE task to trauma-exposed individuals with and without a PTSD diagnosis to provide more nuanced differences in belief updating. Specifically, such studies may assess whether and how updating difficulties predict specific symptom clusters (i.e. re-experiencing, intrusion and avoidance). Finally, it should be emphasized that our approach to combine sample sizes across studies is highly exploratory and findings need to be regarded as preliminary. Adopting a sequential Bayesian testing approach (Schönbrodt et al., 2017) may help future studies to overcome the limitations of our approach while being able to adaptively increase the sample size to detect potentially existing effects. This consideration underscores the importance of further research to validate and refine our findings.

If confirmed by prospective, longitudinal research, the current findings may bear several practical clinical implications. One possibility is the development of interventions specifically designed to optimize belief updating in individuals prior to or immediately after trauma exposure, with the aim of potentially reducing the likelihood of symptom development (Kube & Rozenkrantz, 2021). While trauma-focused psychotherapies already target belief updating processes, there may be room for interventions that specifically focus on enhancing this aspect. In this regard, expectation-optimization interventions could be explored as a supplementary approach to existing trauma-focused psychotherapies for individuals with PTSD, with the potential to enhance treatment outcomes (Michael et al., 2019). These interventions could be particularly beneficial in addressing trauma-related beliefs that are resistant to change and contribute to feelings of shame and guilt. Such beliefs, like ‘I shouldn't have gone back to the apartment with the abuser; it was my fault that it happened’ or ‘Had I only done something in the situation, then my mother wouldn't have been killed,’ often interfere with successful event processing during exposure sessions (Müller-Engelmann et al., 2019). However, further research is required to establish the effectiveness and feasibility of these potential clinical implications.

## Supplementary Material

Supplementary Table 1_Sample Characteristics.docx

## Data Availability

The data that support the findings of this study will be made available via OSF upon publication.

## References

[CIT0001] Amir, N., Coles, M. E., & Foa, E. B. (2002). Automatic and strategic activation and inhibition of threat-relevant information in posttraumatic stress disorder. *Cognitive Therapy and Research*, *26*(5), 645–655. 10.1023/A:1020309326976

[CIT0002] APA. (2013). American Psychiatric Association, 2013. Diagnostic and statistical manual of mental disorders (5th ed.). In *American Journal of Psychiatry*.

[CIT0003] Barr, N., Pennycook, G., Stolz, J. A., & Fugelsang, J. A. (2015). Reasoned connections: A dual-process perspective on creative thought. *Thinking & Reasoning*, *21*(1), 61–75. 10.1080/13546783.2014.895915

[CIT0004] Benjet, C., Bromet, E., Karam, E. G., Kessler, R. C., McLaughlin, K. A., Ruscio, A. M., Shahly, V., Stein, D. J., Petukhova, M., Hill, E., Alonso, J., Atwoli, L., Bunting, B., Bruffaerts, R., Caldas-de-Almeida, J. M., de Girolamo, G., Florescu, S., Gureje, O., Huang, Y., … Koenen, K. C. (2016). The epidemiology of traumatic event exposure worldwide: Results from the World Mental Health Survey Consortium. *Psychological Medicine*, *46*(2), 327–343. 10.1017/S0033291715001981 26511595 PMC4869975

[CIT0005] Bernardi, J., Engelbrecht, A., & Jobson, L. (2019). The impact of culture on cognitive appraisals: Implications for the development, maintenance, and treatment of posttraumatic stress disorder. *Clinical Psychologist*, *23*(2), 91–102. 10.1111/cp.12161

[CIT0006] Blanco, C., Hoertel, N., Wall, M. M., Franco, S., Peyre, H., Neria, Y., Helpman, L., & Limosin, F. (2018). Toward understanding sex differences in the prevalence of posttraumatic stress disorder: Results from the national epidemiologic survey on alcohol and related conditions. *The Journal of Clinical Psychiatry*, *79*(2), 16m11364. 10.4088/JCP.16m11364 29659210

[CIT0007] Blevins, C. A., Weathers, F. W., Davis, M. T., Witte, T. K., & Domino, J. L. (2015). The posttraumatic stress disorder checklist for DSM-5 (PCL-5): Development and initial psychometric evaluation. *Journal of Traumatic Stress*, *28*(6), 489–498. 10.1002/jts.22059 26606250

[CIT0008] Bovin, M. J., Marx, B. P., Weathers, F. W., Gallagher, M. W., Rodriguez, P., Schnurr, P. P., & Keane, T. M. (2016). Psychometric properties of the PTSD checklist for diagnostic and statistical manual of mental disorders-fifth edition (PCL-5) in veterans. *Psychological Assessment**,* *28* *(**11**)*, 1379–1391. 10.1037/pas0000254 26653052

[CIT0009] Böschen, I. (2023). Changes in methodological study characteristics in psychology between 2010–2021. *PLoS One**,* *18* *(**5**)*, e0283353. 10.1371/journal.pone.0283353 37163505 PMC10171647

[CIT0010] Brown, L. A., Belli, G. M., Asnaani, A., & Foa, E. B. (2019). A review of the role of negative cognitions about oneself, others, and the world in the treatment of PTSD. *Cognitive Therapy and Research*, *43*(1), 143–173. 10.1007/s10608-018-9938-1

[CIT0011] Bryant, R. A. (2019). Post-traumatic stress disorder: A state-of-the-art review of evidence and challenges. *World Psychiatry*, *18*(3), 259–269. 10.1002/wps.20656 31496089 PMC6732680

[CIT0012] Campitelli, G., & Gerrans, P. (2014). Does the cognitive reflection test measure cognitive reflection? A mathematical modeling approach. *Memory & Cognition*, *42*(3), 434–447. 10.3758/s13421-013-0367-9 24132723

[CIT0013] Chmielewski, M., & Kucker, S. C. (2020). An MTurk crisis? Shifts in data quality and the impact on study results. *Social Psychological and Personality Science*, *11*(4), 464–473. 10.1177/1948550619875149

[CIT0014] Croft, J., Teufel, C., Heron, J., Fletcher, P. C., David, A. S., Lewis, G., Moutoussis, M., FitzGerald, T. H., Linden, D. E., Thompson, A., & Jones, P. B. (2022). A computational analysis of abnormal belief updating processes and their association with psychotic experiences and childhood trauma in a UK birth cohort. *Biological Psychiatry: Cognitive Neuroscience and Neuroimaging*, *7*(7), 725–734. 10.1016/j.bpsc.2021.12.007 34954139 PMC9259502

[CIT0015] DePrince, A. (2005). Social cognition and revictimization risk. *Journal of Trauma & Dissociation*, *6*(1), 125–141. 10.1300/J229v06n01_08 16150689

[CIT0016] Diamond, G. M., Lipsitz, J. D., Fajerman, Z., & Rozenblat, O. (2010). Ongoing traumatic stress response (OTSR) in Sderot, Israel. *Professional Psychology: Research and Practice*, *41*(1), 19–25. 10.1037/a0017098

[CIT0017] Ehlers, A., & Clark, D. M. (2000). A cognitive model of posttraumatic stress disorder. *Behaviour Research and Therapy*, *38*(4), 319–345. 10761279 10.1016/s0005-7967(99)00123-0

[CIT0018] Elwood, L. S., Williams, N. L., Olatunji, B. O., & Lohr, J. M. (2007). Interpretation biases in victims and non-victims of interpersonal trauma and their relation to symptom development. *Journal of Anxiety Disorders*, *21*(4), 554–567. 10.1016/j.janxdis.2006.08.006 16963221

[CIT0019] Everaert, J., Bronstein, M. V., Cannon, T. D., & Joormann, J. (2018). Looking through tinted glasses: Depression and social anxiety are related to both interpretation biases and inflexible negative interpretations. *Clinical Psychological Science*, *6*(4), 517–528. 10.1177/2167702617747968

[CIT0020] Faul, F., Erdfelder, E., Lang, A.-G., & Buchner, A. (2007). G∗Power 3: A ﬂexible statistical power analysis program for the social, behavioral, and biomedical sciences. *Behavior Research Methods*, *39*(2), 175–191. 10.3758/BF03193146 17695343

[CIT0021] Feldmann, M., Kube, T., Rief, W., & Brakemeier, E. L. (2023). Testing Bayesian models of belief updating in the context of depressive symptomatology. *International Journal of Methods in Psychiatric Research*, *32*(2), e1946. 36114811 10.1002/mpr.1946 PMC10242189

[CIT0022] Flory, J. D., & Yehuda, R. (2015). Comorbidity between post-traumatic stress disorder and major depressive disorder: Alternative explanations and treatment considerations. *Dialogues in Clinical Neuroscience*, *17*(2), 141–150. 10.31887/DCNS.2015.17.2/jflory 26246789 PMC4518698

[CIT0023] Foa, E. B., & Kozak, M. J. (1986). Emotional processing of fear: Exposure to corrective information. *Psychological Bulletin*, *99*(1), 20–35. 10.1037/0033-2909.99.1.20 2871574

[CIT0024] Foa, E. B., & McLean, C. P. (2016). The efficacy of exposure therapy for anxiety-related disorders and its underlying mechanisms: The case of OCD and PTSD. *Annual Review of Clinical Psychology*, *12*(1), 1–28. 10.1146/annurev-clinpsy-021815-093533 26565122

[CIT0025] Frederick, S. (2005). Cognitive reflection and decision making. *Journal of Economic Perspectives**,* *19*(4), 25–42. 10.1257/089533005775196732

[CIT0026] Gobin, R. L., & Freyd, J. J. (2009). Betrayal and revictimization: Preliminary findings. *Psychological Trauma: Theory, Research, Practice, and Policy**,* *1*(3), 242–257. 10.1037/a0017469

[CIT0027] Haim-Nachum, S., & Levy-Gigi, E. (2019). A chink in the armor: The influence of training on generalization learning impairments after viewing traumatic stimuli. *Cognition**,* *193*, 104021. 10.1016/j.cognition.2019.104021 31419620

[CIT0028] Haim-Nachum, S., & Levy-Gigi, E. (2021). To be or not to be flexible: Selective impairments as a means to differentiate between depression and PTSD symptoms. *Journal of Psychiatric Research*, *136*, 366–373. 10.1016/j.jpsychires.2021.02.015 33639329

[CIT0029] Herzog, P., Kaiser, T., Rief, W., Brakemeier, E. L., & Kube, T. (2021). Assessing dysfunctional expectations in posttraumatic stress disorder: Development and validation of the posttraumatic expectations scale (PTES). *Assessment*, *30*(4), 1285–1301. 10.1177/10731911221089038 PMC11951371 35549727

[CIT0030] Horn, S. R., & Feder, A. (2018). Understanding resilience and preventing and treating PTSD. *Harvard Review of Psychiatry*, *26*(3), 158–174. 10.1097/HRP.0000000000000194 29734229

[CIT0031] Howlett, J. R., Bomyea, J., Harlé, K. M., & Simmons, A. N. (2021). Symptoms of posttraumatic stress disorder are associated with exaggerated neural response to surprising errors. *Journal of Traumatic Stress*, *34*(1), 172–181. 10.1002/jts.22595 33025689

[CIT0032] Kees, J., Berry, C., Burton, S., & Sheehan, K. (2017). An analysis of data quality: Professional panels, student subject pools, and Amazon's Mechanical Turk. *Journal of Advertising*, *46*(1), 141–155. 10.1080/00913367.2016.1269304

[CIT0033] Kessler, R. C., Aguilar-Gaxiola, S., Alonso, J., Benjet, C., Bromet, E. J., Cardoso, G., Degenhardt, L., de Girolamo, G., Dinolova, R. V., Ferry, F., & Florescu, S. (2017). Trauma and PTSD in the WHO world mental health surveys. *European Journal of Psychotraumatology*, *8*(sup5), 1353383. 10.1080/20008198.2017.1353383 29075426 PMC5632781

[CIT0034] Kleim, B., Grey, N., Wild, J., Nussbeck, F. W., Stott, R., Hackmann, A., Clark, D. M., & Ehlers, A. (2013). Cognitive change predicts symptom reduction with cognitive therapy for posttraumatic stress disorder. *Journal of Consulting and Clinical Psychology*, *81*(3), 383–393. 10.1037/a0031290 23276122 PMC3665307

[CIT0035] Kraemer, H. C., Morgan, G. A., Leech, N. L., Gliner, J. A., Vaske, J. J., & Harmon, R. J. (2003). Measures of clinical significance. *Journal of the American Academy of Child & Adolescent Psychiatry*, *42*(12), 1524–1529. 10.1097/00004583-200312000-00022 14627890

[CIT0036] Krause, J. S., Reed, K. S., & McArdle, J. J. (2010). Factor structure and predictive validity of somatic and nonsomatic symptoms from the patient health questionnaire-9: A longitudinal study after spinal cord injury. *Archives of Physical Medicine and Rehabilitation*, *91*(8), 1218–1224. 10.1016/j.apmr.2010.04.015 20684902

[CIT0037] Kroenke, K., Spitzer, R. L., & Williams, J. B. (2001). The PHQ-9: Validity of a brief depression severity measure. *Journal of General Internal Medicine*, *16*(9), 606–613. 10.1046/j.1525-1497.2001.016009606.x 11556941 PMC1495268

[CIT0100] Kube, T. (2023). Biased belief updating in depression. *Clinical Psychology Review*, *103*, 102298. 37290245 10.1016/j.cpr.2023.102298

[CIT0038] Kube, T., Berg, M., Kleim, B., & Herzog, P. (2020). Rethinking post-traumatic stress disorder–A predictive processing perspective. *Neuroscience & Biobehavioral Reviews*, *113*, 448–460. 10.1016/j.neubiorev.2020.04.014 32315695

[CIT0039] Kube, T., Elssner, A. C., & Herzog, P. (2023). The relationship between multiple traumatic events and the severity of posttraumatic stress disorder symptoms – evidence for a cognitive link. *European Journal of Psychotraumatology*, *14*(1), 2165025. 10.1080/20008066.2023.2165025 PMC9879173 37052097

[CIT0041] Kube, T., Kirchner, L., Lemmer, G., & Glombiewski, J. A. (2022). How the discrepancy between prior expectations and new information influences expectation updating in depression—the greater, the better? *Clinical Psychological Science*, *10*(3), 430–449. 10.1177/21677026211024644

[CIT0042] Kube, T., & Rozenkrantz, L. (2021). When beliefs face reality: An integrative review of belief updating in mental health and illness. *Perspectives on Psychological Science*, *16*(2), 247–274. 10.1177/1745691620931496 32818386

[CIT0043] Lahad, M., & Leykin, D. (2010). Ongoing exposure versus intense periodic exposure to military conflict and terror attacks in Israel. *Journal of Traumatic Stress*, *23*(6), 691–698. 10.1002/jts.20583 21171129

[CIT0044] Lakens, D., Scheel, A. M., & Isager, P. M. (2018). Equivalence testing for psychological research: A tutorial. *Advances in Methods and Practices in Psychological Science*, *1*(2), 259–269. 10.1177/2515245918770963

[CIT0045] Lazarov, A., Suarez-Jimenez, B., Tamman, A., Falzon, L., Zhu, X., Edmondson, D. E., & Neria, Y. (2019). Attention to threat in posttraumatic stress disorder as indexed by eye-tracking indices: A systematic review. *Psychological Medicine*, *49*(5), 705–726. 10.1017/S0033291718002313 30178728 PMC6399079

[CIT0046] Levy-Gigi, E., & Richter-Levin, G. (2014). The hidden price of repeated traumatic exposure. *Stress*, *17*(4), 343–351. 10.3109/10253890.2014.923397 24810272

[CIT0047] Levy-Gigi, E., Richter-Levin, G., & Kéri, S. (2014). The hidden price of repeated traumatic exposure: Different cognitive deficits in different first-responders. *Frontiers in Behavioral Neuroscience**,* *8*, 281. 10.3389/fnbeh.2014.00281 25191237 PMC4138485

[CIT0048] Linson, A., & Friston, K. J. (2019). Reframing PTSD for computational psychiatry with the active inference framework. *Cognitive Neuropsychiatry*, *24*(5), 347–368. 10.1080/13546805.2019.1665994 31564212 PMC6816477

[CIT0049] Michael, T., Schanz, C. G., Mattheus, H. K., Issler, T., Frommberger, U., Köllner, V., & Equit, M. (2019). Do adjuvant interventions improve treatment outcome in adult patients with posttraumatic stress disorder receiving trauma-focused psychotherapy? A systematic review. *European Journal of Psychotraumatology*, *10*(1), 1634938. 10.1080/20008198.2019.1634938 31489131 PMC6711134

[CIT0050] Müller-Engelmann, M., Schreiber, C., Kümmerle, S., Heidenreich, T., Stangier, U., & Steil, R. (2019). A trauma-adapted mindfulness and loving-kindness intervention for patients with PTSD after interpersonal violence: A multiple-baseline study. *Mindfulness*, *10*(6), 1105–1123. 10.1007/s12671-018-1068-z

[CIT0051] Olff, M. (2017). Sex and gender differences in post-traumatic stress disorder: An update. *European Journal of Psychotraumatology**,* *8*(sup4), 1351204. 10.1080/20008198.2017.1351204

[CIT0052] Ossola, P., Garrett, N., Sharot, T., & Marchesi, C. (2020). Belief updating in bipolar disorder predicts time of recurrence. *Elife*, *9*. 10.7554/eLife.58891 PMC7655098 33168133

[CIT0053] Primi, C., Morsanyi, K., Donati, M. A., & Chiesi, F. (2014). Item response theory analysis of the cognitive reflection test: Testing the psychometric. *In Issue: Proceedings of the Annual Meeting of the Cognitive Science Society*, *36*, 2799–2804.

[CIT0054] Richardson, E. J., & Richards, J. S. (2008). Factor structure of the PHQ-9 screen for depression across time since injury among persons with spinal cord injury. *Rehabilitation Psychology*, *53*(2), 243–249. 10.1037/0090-5550.53.2.243

[CIT0055] Sayed, S., Iacoviello, B. M., & Charney, D. S. (2015). Risk factors for the development of psychopathology following trauma. *Current Psychiatry Reports*, *17*(8), 1–7. 10.1007/s11920-015-0612-y 26206108

[CIT0056] Schönbrodt, F. D., Wagenmakers, E. J., Zehetleitner, M., & Perugini, M. (2017). Sequential hypothesis testing with Bayes factors: Efficiently testing mean differences. *Psychological Methods**,* *22* *(**2**)*, 322–339. 10.1037/met0000061 26651986

[CIT0057] Sopp, M. R., Haim-Nachum, S., Wirth, B. E., Bonanno, G. A., & Levy-Gigi, E. (2022). Leaving the door open: Trauma, updating, and the development of PTSD symptoms. *Behaviour Research and Therapy*, *154*, 104098. 10.1016/j.brat.2022.104098 35617768

[CIT0058] Spielberger, C. D. (1989). *State-Trait Anxiety Inventory: Bibliography* (2nd ed.). Consulting Psychologists Press.

[CIT0059] Spielberger, C. D., Gorsuch, R. L., Lushene, R. E., Vagg, P. R., & Jacobs, G. A. (1983). Manual for 22 the State-Trait Anxiety Inventory (STAI) Form Y. Palo Alto, CA Consulting 23 Psychologists Press.

[CIT0060] Ter Heide, F. J. J., Sleijpen, M., & van der Aa, N. (2017). Posttraumatic world assumptions among treatment-seeking refugees. *Transcultural Psychiatry*, *54*(5–6), 824–839. 10.1177/1363461517741811 29226792

[CIT0061] Toplak, M. E., West, R. F., & Stanovich, K. E. (2011). The Cognitive Reflection Test as a predictor of performance on heuristics-and-biases tasks. *Memory & Cognition*, *39*(7), 1275–1289. 10.3758/s13421-011-0104-1 21541821

[CIT0062] Weathers, F. W., Blake, D. D., Schnurr, P. P., Kaloupek, D. G., Marx, B. P., & Keane, T. M. (2013). The life events checklist for DSM-5 (LEC-5). Instrument available from the National Center for PTSD at www.ptsd.va.gov.

[CIT0063] Weathers, F. W., Litz, B. T., Keane, T. M., Palmieri, P. A., Marx, B. P., & Schnurr, P. P. (2013). The PTSD Checklist for DSM-5 (PCL-5).

[CIT0064] White, M., McManus, F., & Ehlers, A. (2008). An investigation of whether patients with post-traumatic stress disorder overestimate the probability and cost of future negative events. *Journal of Anxiety Disorders*, *22*(7), 1244–1254. 10.1016/j.janxdis.2008.01.004 18316175 PMC2956503

[CIT0065] Wilkinson, S., Dodgson, G., & Meares, K. (2017). Predictive processing and the varieties of psychological trauma. *Frontiers in Psychology*, *8*, 1840. 10.3389/fpsyg.2017.01840 29089916 PMC5651022

[CIT0066] Woodward, T. S., Moritz, S., Cuttler, C., & Whitman, J. C. (2006). The contribution of a cognitive bias against disconfirmatory evidence (BADE) to delusions in schizophrenia. *Journal of Clinical and Experimental Neuropsychology*, *28*(4), 605–617. 10.1080/13803390590949511 16624787

[CIT0067] Woud, M. L., Kleim, B., & Cwik, J. C. (2019). Editorial for the special issue on negative appraisals in trauma: Current status and future directions for research. *Cognitive Therapy and Research*, *43*(1), 139–142. 10.1007/s10608-018-09992-5

