# Supplementary material for: Does disconfirmatory evidence shape safety-and danger-related beliefs of trauma-exposed individuals?
Source: Eur J Psychotraumatol. 2024 Apr 16;15(1):2335788. doi: 10.1080/20008066.2024.2335788 (PMC11022916; doi:10.1080/20008066.2024.2335788)
Supplement: Supplementary Table 1_Sample Characteristics.docx [file ZEPT_A_2335788_SM7910.docx]

**Supplementary Table 1A**

*Demographic and psychometric characteristics for TE and NT individuals. Means and (SD)*

| **Variables** | **Trauma-exposed**  **(n =180)** | **Non-trauma exposed**  **(n = 41)** | ***P*-value** |
| --- | --- | --- | --- |
| Age (years) | 47.11 (14.79) | 44.12 (15.32) | .25 |
| Female/Male | 92/88 | 23/18 | .57 |
| Education (years) | 15.04 (2.71) | 14.46 (2.55) | .21 |
| PCL-5 | 11.39 (13.24) | --- | .00 |
| PHQ-9 | 4.55 (4.5) | 2.71 (3.59) | .01 |
| LEC-5 | 4.14 (2.58) | 0 | .00 |
| STAI State | 37.33 (12.4) | 33.83 (9.12) | .04 |
| STAI Trait | 36.63 (12.15) | 33.24 (10.98) | .10 |
| TR-BADE_DD | 6.94 (3.67) | 6.21 (3.33) | .24 |
| TR-BADE_DS | 8.86 (4.06) | 8.1 (4.58) | .29 |
| CRT | 0.67 (0.92) | 0.46 (0.92) | .19 |

*N* = 221

Note. The values for Female/Male and households represent frequencies. PCL-5 = PTSD checklist for DSM-V; PHQ-9 = depressive symptoms; LEC-5 = life events checklist; STAI – anxiety scores; TR-BADE_DD = disconfirming danger scenarios; TR-BADE_DS = disconfirming safety scenarios; CRT = Cognitive Reflection Test.

**Supplementary Table 1B**

*Demographic and psychometric characteristics for TE individuals.*

| **Variables** | **Means and (*SD)*** |
| --- | --- |
| Age (years) | 48.33 (14.33) |
| Female/Male | 82/81 |
| Education (years) | 15.68 (2.81) |
| PCL-5 | 12.34 (13.61) |
| PHQ-9 | 5.47 (4.51) |
| LEC-5 | 4.42 (2.7) |
| TR-BADE_DD | 6.83 (3.62) |
| TR-BADE_DS | 9.19 (4.39) |

*N* = 163

Note. The values for Female/Male and households represent frequencies. PCL-5 = PTSD checklist for DSM-V; PHQ-9 = depressive symptoms; LEC-5 = life events checklist; TR-BADE_DD = disconfirming danger scenarios; TR-BADE_DS = disconfirming safety scenarios.
